# Health Belief Model in studies of influenza vaccination among health care workers

**DOI:** 10.47626/1679-4435-2022-839

**Published:** 2023-08-08

**Authors:** Suellen Bittencourt Silva, Fernanda de Oliveira Souza, Paloma de Sousa Pinho, Deisy Vital Santos

**Affiliations:** 1 Centro de Ciências da Saúde, Universidade Federal do Recôncavo da Bahia, Santo Antônio de Jesus, BA, Brazil

**Keywords:** influenza vaccines, health personnel, health belief model, vacinas contra *influenza*, pessoal de saúde, modelo de crenças de saúde

## Abstract

Vaccines prevent numerous infectious diseases, including influenza. Despite their significant contribution to controlling influenza, vaccine coverage against this disease varies among health care workers. The Health Belief Model could thus help to understand the reasons why these workers accept (or not) the immunobiological. The aim of this study was to describe the main results of research performed on influenza vaccination among health care workers using the Health Belief Model. This is an integrative literature review. Data search took place in October 2020 in the PubMed database, with the following descriptors: “influenza vaccine“; “health professionals”; “Health Belief Model,” and their synonyms. Eleven studies were included in this review. The main dimensions of the model (susceptibility, severity, benefits, and barriers) were more explored by the studies, and self-efficacy was the least studied dimension. Moreover, we observed a relationship between the theory’s dimensions (susceptibility, severity, benefits, barriers, cues to action, and self-efficacy) and influenza vaccination in health care workers. In conclusion, this review identified profiles of beliefs for each dimension of the Health Belief Model, which has traditionally been an ally for determining refusal or acceptance of the influenza vaccine among health care workers.

## Abstract (pt)

As vacinas previnem inúmeras doenças infecciosas, inclusive a *influenza*. Mesmo diante da sua significativa contribuição para o controle da enfermidade, as coberturas vacinais contra *influenza* entre os trabalhadores de saúde são variáveis. Dessa forma, o modelo de crenças em saúde pode auxiliar na compreensão dos motivos que levam esses trabalhadores a aceitarem ou não o imunobiológico. O objetivo deste estudo foi descrever os principais resultados de pesquisas sobre vacinação contra *influenza* entre trabalhadores de saúde utilizando o modelo de crenças em saúde. Tratou-se de uma revisão integrativa da literatura. A busca de dados ocorreu em outubro de 2020 na base de dados PubMed, com os descritores: “influenza vaccine”; “health professionals”; e “Health Belief Model” e seus sinônimos. Foram incluídos 11 artigos nesta revisão. Identificou-se que as principais dimensões do modelo (suscetibilidade, gravidade, benefícios e barreiras) foram mais exploradas pelos artigos, e a dimensão autoeficácia foi a menos estudada. Além disso, existe uma relação entre as dimensões da teoria (suscetibilidade, gravidade, benefícios, barreiras, estímulos para ação e autoeficácia) e a vacinação contra a *influenza* em trabalhadores da saúde. Conclui-se que, através da revisão proposta, pôde-se identificar perfis de crenças para cada dimensão do modelo de crenças em saúde que, tradicionalmente, vem servindo como aliado para determinar recusa ou aceitação da vacina contra *influenza* entre trabalhadores de saúde.

## INTRODUCTION

To this day, vaccination is the most effective and low-cost method for preventing numerous infectious diseases. Since their discovery, they have been saving countless lives and contributing to improvements in health and well-being worldwide.^1^ The World Health Organization^2^ recommends immunization against influenza primarily for the following target populations: pregnant women, children aged 6 to 59 months, older adults, individuals with chronic diseases, and health care workers.

The influenza virus is capable of producing a flu-like syndrome with an epidemic character and high morbidity, presenting high hospitalization rates among older adults and people with chronic diseases. Its transmission occurs mainly through droplets released by infected individuals when talking, coughing, or sneezing, and also through contact with one’s secretions; immunization is the best preventive st rateg y.^3,4^

Therefore, influenza vaccination in health care workers presents the following benefits: decreased infection rates among workers; decreased possibility of contaminating the users of health systems due to reduced transmission at health care facilities; reduced probability of infecting the workers’ family members; and reduced absenteeism, which would benefit employers.^4,5^

Despite its significant contribution to controlling influenza, vaccine coverage against this disease among health care workers varies. Notably, average coverage rates are seen in Europe (29.5%)^6^; in Israel, vaccine coverage is 42%^7^; in the United States, 78.4%^8^; in Brazil, 91.25%.^9^

Some studies indicate aspects that contribute to greater adherence to influenza vaccination, such as: offering vaccines free of charge; with convenient access, including at health facilities; knowledge on the disease and production process of immunobiologicals; belief in vaccine effectiveness; and risk perception.^5,10^

In the 1950s, the Health Belief Model (HBM) was formulated by social psychologists based on two theoretical sources: the theory of cues to action (CTA) and the cognitive theory, for exploring different health-related behaviors. According to the HBM, for an individual to act preemptively, he or she needs to believe that: a) he or she is susceptible to an undesirable disease or condition; b) its occurrence presents severity to some aspect of his or her life; c) performing a prophylactic intervention is effective for reducing disease susceptibility or severity (benefits); and d) the preventive action does not imply in too many barriers.^11,12^

The HBM was further reformulated by Rosenstock et al.,^13^ when two other categories were included: e) cues to action; and f) self-efficacy or health motivation.^11,12^ Nowadays, HBM is useful for understanding influenza vaccine acceptance or not among different categories of workers in the health care sector.^14^

This model was used both in the study of the reasons why health care workers accept influenza vaccines (or not)^15^ and in the development of interventions for improving adherence.^16^

Considering this reality, the aim of this study was to describe the main results of research performed on influenza vaccination among health care workers using the HBM.

## METHODS

This integrative review was based on the methodological assumptions of Whittemore & Knafl,^17^ being divided into the following stages: problem identification, literature search, data evaluation, data analysis, and presentation of the integrative review.

For identifying the problem, this study had the following research question: “What are the results presented by studies on influenza vaccination in health care workers through the use of the HBM?”

For answering this question, our search was performed in October 2020 in the National Library of Medicine (PubMed) database with the following Medical Subject Headings/Descritores em Ciências da Saúde (MeSH/DeCS): “influenza vaccine”; “health professionals”; “Health Belief Model” and their synonyms (in English). We used the Boolean operator AND, aiming to reach the content of all established descriptors.

The following inclusion criteria were employed: original articles with full text available, regarding the HBM applied to influenza vaccination in health care workers. We highlight that no restrictions of for time since publication were established, in order to broaden the number of studies being reviewed.

**Figure 1:**
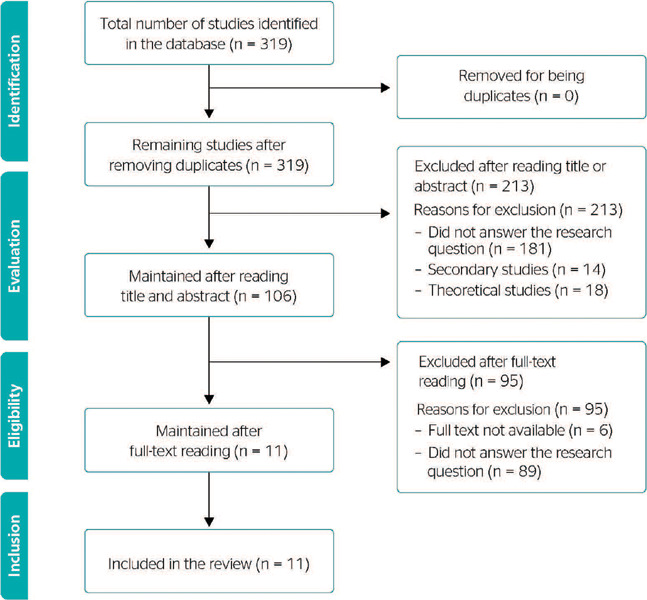
Flowchart of the article selection process for the integrative review, according to the *Preferred Reporting Items for Systematic Reviews and Meta-Analyses* (PRISMA) checklist.^18^

In the first search, we selected 319 studies. Therefore, we used the Rayyan tool for evaluating the obtained data.^18^ Considering the inclusion criteria and after reading the titles and abstracts, we excluded 213 studies and a total of 106 articles remained. There were no duplicates; however, 6 studies could not be fully retrieved. The full text of these 100 studies was then analyzed and, after applying the aforementioned criteria, 11 studies were selected for this review, as shown in Figure 1.

The articles were classified according to the level of evidence proposed by the Agency for Healthcare Research and Quality (AHRQ).^19^ This categorization qualifies articles into six levels, as follows: level 1 – meta-analysis of controlled studies; level 2 – study with experimental design; level 3 – study with a quasi-experimental design, such as a non-randomized or case-control study; level 4 – study with a non-experimental design, such as descriptive correlational and qualitative research or case studies; level 5 – case report or data obtained systematically; and level 6 – expert opinions.

For data collection and organization, we used an instrument constructed by the authors with two sets of questions: the first one comprised items related to article identification (title, authors, and year of publication), place (country), research participants, and type of influenza; and the second contained the identification of HBM dimensions and the main conclusions regarding them.

Finally, we presented the results, with a synthesis of findings about HBM on influenza vaccination among health care workers, which were systematized into two categories: the main HBM dimensions and the two new HBM dimensions.

## RESULTS AND DISCUSSION

Out of the 11 selected articles (Table 1), 10 studies used a quantitative approach^20,21,22,23,24,25,26,27,28,29^ and only 1 employed a qualitative method.^30^ Considering the levels of evidence, all studies were classified as level 4. The articles were published between 2005 and 2020, but mainly on 2019, which presented three publications.^21,22,29^ The sample sizes of the studies varied between 30 and 3,971 workers, with a mean value of 1,328 and a median value of 601.

**Table 1:** Characterization of the studies included in the integrative review

Identification	Title	Authorship (year)	Country	Professional category studied	Sample size	Type of influenza
A1^20^	The Health Belief Model in predicting healthcare workers’ intention for influenza vaccine uptake in Jordan	Alhalaseh L, Fayoumi H, Khalil B (2020)	Jordan	Physicians and medical staff (nurses, clinical pharmacists, physical therapists, occupational therapists, and other technicians with direct contact with patients)	477 workers	Pandemic and seasonal
A2^21^	Fatores associados à aceitação da vacina influenza entre trabalhadores de saúde: conhecimento, attitude e prática	De Souza TP, Lobão WM, Santos CAST, Almeida MCC, Júnior EDM (2019)	Brazil	Nurses, practical nurses, physicians, physical therapists, dietitians, laboratory technicians, cleaning workers, medical office assistants, and cafeteria workers.	755 workers	Seasonal*
A3^22^	Factors influencing seasonal influenza vaccination uptake among health care workers in an adult tertiary care hospital in Singapore: a cross-sectional survey	Kyaw WM, Chow A, Hein AA, Lee LT, Leo YS, Ho HJ (2019)	Singapore	Administrative staff, allied health staff (pharmacists, dietitians, and clinical research coordinators), ancillary staff (administrative assistants, health attendants, and technicians), and medical and nursing staff.	3,873 workers	Seasonal
A4^29^	Multi-centre study on cultural dimensions and perceived attitudes of nurses towards influenza vaccination uptake.	Kwok KO, Li KK, Lee SS, Chng PHY, Wei VWI, Ismail NH, et al. (2019)	Hong Kong, Singapore, and Brunei	In Hong Kong, registered nurses, enrolled nurses, and nursing students/interns; in Singapore, registered and enrolled nurses; and in Brunei, registered nurses	3,971 workers	Seasonal
A5^23^	Intention to receive the seasonal influenza vaccine among nurses working in a long-term care facility	Shahar I, Mendelson G, Ben Natan M (2017)	Israel	Nurses	150 nurses	Seasonal
A6^25^	External cues to action and influenza vaccination among post-graduate trainee physicians in Toronto, Canada	Nowrouzi-Kia B, McGeer A (2014)	Canada	Trainee physicians registered at a graduate program	935 workers	Pandemic and seasonal
A7^26^	Predicting influenza vaccination uptake among health care workers: what are the key motivators?	Corace K, Prematunge C, McCarthy A, Nair RC, Roth V, Hayes T, et al. (2013)	Canada	Nurses, physicians, allied and administrative staff, health care, research and laboratory, and facilities and logistics technicians, and other nonclinical workers	3,275 people	Pandemic and seasonal
A8^27^	Predictors of influenza vaccination among emergency medical services personnel	Hubble MW, Zontek TL, Richards ME (2011)	United States of America	Workers at emergency health care units involved in direct patient care	601 workers	Pandemic, seasonal, and avian flu
A9^24^	Factors affecting nurses’ decision to get the flu vaccine	Shahrabani S, Benzion U, Yom Din G (2009)	Israel	Undergraduate and graduate nursing students/interns	299 participants	Seasonal†
A10^30^	Attitudes of nurses in Greece towards influenza vaccination	Raftopoulos V (2008)	Greece	Registered nurses	30 nurses	Pandemic and avian flu
A11^28^	Actions and beliefs related to hepatitis B and influenza immunization among registered nurses in Texas	McEwen M, Farren E (2005)	United States of America	Registered nurses	246 nurses	Seasonal†

* Although not specified by the author, data were collected in 2015, referring to the 2014 campaign, which employed vaccines against seasonal influenza only.

† Although not specified by the author, data were collected between November 2005 and January 2006, when only vaccines against seasonal influenza were employed.

‡Although not specified by the author, data were collected in 2004, when only vaccines against seasonal influenza were employed.

As to the country where the studies were conducted, one of them was carried out in Jordan,^20^ one in Brazil,^21^ one in Singapore,^22^ two in Israel,^23,24^ two in Canada,^25,26^ two in the United States,^27,28^ and one in Greece.^30^ In addition, one study had a multicenter design (Hong Kong, Singapore, and Brunei).^29^

Different categories of workers were presented in the studies, such as: nurses participated in nine studies,^20,21,22,23,24,26,28,27,28,29,30^ physicians were present in five of them,^20,21,22,25,26^ ancillary staff (technical, cleaning, and administrative) were present in four publications,^20,21,22,26^ workers of emergency medical services were studied in only one article,^27^ and other professionals such as pharmacists, physical therapists, and dietitians were studied in four articles.^20,21,22,26^

Regarding the type of influenza, most articles considered the seasonal^20,22,23,25,26,27,29^ and pandemic types.^20,25,26,27,30^ Some studies also mentioned avian flu,^27,30^ and other three articles did not specify the flu type; however, considering the date of data collection, we may infer that it was seasonal influenza.^21,24,28^

### MAIN HBM DIMENSIONS: SUSCEPTIBILITY, SEVERITY, BENEFITS, AND BARRIERS

Most articles (82%) presented in this review approached the four main dimensions of the HBM. However, one of them did not analyze the perceived barriers dimension,^29^ and another one did not present perceived severity^28^ (Table 2).

**Table 2:** Identification of the four main dimensions of the Health Belief Model among the 11 studies

Study	Susceptibility	Severity	Benefits	Barriers
A1^20^	The workers who intended to get vaccinated had a significantly higher probability of recognizing the increase in susceptibility of their ill patients, in addition to their own risk.	The participants recognized the severity of the flu and its complications.	The group of workers who intended to receive the vaccine endorsed its benefits.	The participants who did not intend to receive the vaccine were more likely to perceive barriers as detrimental.
A2^21^	The knowledge that the influenza vaccine does not provide protection for many years was associated with vaccination, possibly because these individuals feel more vulnerable to influenza.	Most participants recognized the severity of the flu.	A statistically significant association with vaccination was observed for recognizing the vaccine as safe and a *borderline* association for acknowledging vaccine efficacy.	Not fearing adverse events was associated with vaccination.
A3^22^	Having family members aged less than 16 years was negatively associated with vaccination, possibly because these young families had other priorities and/or believed they were not at risk of contracting the flu, since they were usually healthy.	The belief in a potential severity of the flu was a predictor of vaccine acceptance.	The belief in vaccine safety was a predictor of vaccine acceptance.	Adverse effects were the main reason for not receiving the vaccine.
A4^29^	An association between susceptibility and vaccine uptake was observed.	No relationship was observed between severity and vaccination.	An association between benefits and vaccine uptake was observed.	-
A5^23^	Significant correlations were observed between susceptibility and willingness to receive the vaccine.	Significant correlations were observed between severity and the willingness to receive the vaccine.	A strong significant correlation was observed between perceived benefits and the intention of getting vaccinated.	A significant correlation was observed between perceived barriers and a low intention of receiving the vaccine.
A6^25^	A significant association was observed between personal susceptibility and receiving the vaccine.	A significant association was observed between perceived severity and vaccination.	The vaccinated interviewees were more likely to agree that the vaccine’s risks outweigh its benefits.	An association was seen between perceived barriers and a reduction in the probability of vaccination.
A7^26^	Vaccinated individuals were more likely to perceive a high personal risk for influenza.	Those who accepted the vaccine were more likely to perceive influenza as personally dangerous.	The confidence in vaccine safety promoted its acceptance.	Unvaccinated workers were more likely to cite barriers to vaccination.
A8^27^	Increased perceived susceptibility was a predictor of vaccine acceptance.	The severity of the flu was one of the reasons for vaccine acceptance.	Favorable beliefs regarding vaccine efficacy were predictors of vaccine acceptance.	One of the reasons for vaccine hesitancy was its unavailability at convenient hours.
A9^24^	Vaccinated workers perceived themselves as more susceptible to the disease.	Vaccinated workers perceived the flu as a more severe disease.	The perceived benefits were predictors of vaccine acceptance.	Vaccinated workers perceived less barriers.
A10^30^	Most participants were not vaccinated, and a large portion did not consider themselves susceptible to the flu.	The participants acknowledged that influenza could cause a global outbreak of severe disease.	The participants acknowledged that vaccinating health care workers could protect patients.	The lack of vaccine availability and distribution were the main reasons for vaccine hesitancy.
A11^28^	One of the reasons for receiving the vaccine was the concern about being at risk of exposure.	-	The perceived benefits were the main contributors for vaccine acceptance.	One of the reasons for vaccine hesitancy were concerns about side effects.

The susceptibility category may be defined as the perceived probability of contracting a disease or an undesired condition.^11,12^ All quantitative studies associated this dimension with higher influenza immunization rates, whereas the conclusion reached by the qualitative study^30^ was that most participants in the focal group were not vaccinated and a large proportion did not consider themselves susceptible to the flu.

The literature review performed by Hofmann et al.^31^ on influenza immunization in health care workers indicated that the lack of perceived susceptibility increased vaccine hesitancy. Moreover, the systematic review by Corace et al.^14^ on interventions based on structures such as HBM for increasing vaccination rates among health care workers also concluded that vaccinated workers were more likely to report that they were susceptible to influenza.

It is important to highlight that, during their work activities, health care workers are exposed to multiple and varied risks related to chemical, physical, biological, psychosocial, and ergonomic hazards, of which biological hazards are the main generators of threats to workers’ health. These workers interact with several pathologies such as the flu, which exposes them to varied health risks and hazards, making them a vulnerable group.^32^

The severity dimension can be defined as the perceived disease severity, that is, the medical and clinical consequences – such as death, disability, and pain – and the social consequences – such as effects on work and social relationships.^11,12^

Regarding the perceived severity, six studies associated it to vaccination^22,23,24,25,26,27^; in two articles, the severity of the flu was acknowledged, but no advances were made in the severity/immunization relation.^20,21^ Kwok et al.^29^ did not find an association between this dimension and vaccination, and participants of the qualitative study conducted by Raftopoulos^30^ indicated that influenza could cause a global outbreak of severe disease, even though most participants were not vaccinated. This way, we note that the association between perceived severity and vaccination was not unanimous.

We highlight that, sometimes, the acknowledgment of one of the dimensions is not translated into action, as it was shown in the qualitative study of a review.^30^ This was also demonstrated in the publication by Hidiroglu et al.,^33^ where a focal group with 33 health care workers in Turkey concluded that, although the participants considered themselves at risk of contracting H1N1, most of them had not received the available immunization.

In addition, other authors also confirmed that perceived severity increased vaccine acceptance: the rapid evidence assessment by Jenkin et al.,^4^ comprising 60 publications and aiming to examine data on the flu in health care environments and impacts of the vaccination of health care workers against influenza; and the mixed study performed in Kenya,^10^ which aimed to evaluate the knowledge, attitudes, and practices on infection and vaccination among health care workers against H1N1 and noticed that health care workers were more likely to accept vaccination when they believed H1N1 could lead to death.

Another dimension analyzed in different publications are the perceived benefits, which may be defined as the belief in the efficacy of a prophylactic intervention in reducing disease susceptibility or severity.^11,12^ The results of this review indicate that authors who used a quantitative methodology associated perceived benefits to influenza vaccination. However, the article by Raftopoulos,^30^ with a qualitative methodology, revealed that the participants of the focal group acknowledged that vaccinating health care workers could protect patients, even though most of them were not vaccinated. The perceived benefits dimension also presented an association in other similar studies.^4,10,14^

It is important to note that health care workers are at greater risk of infections by influenza when compared to the general population due to their close contact with infected patients at health care facilities.^34^ Nevertheless, the vaccination of health care workers brings, as benefits, reductions in morbidity and absenteeism related to influenza in these workers, contributing to a safer work activity.^5^

Perceived barriers can be defined as potentially negative aspects of the preventive action, such as the cost and pain.^11,12^ All studies that adopted the quantitative method associated perceived barriers with lower immunization rates, and the qualitative^30^ study noticed that the lack of vaccine availability and distribution were the main reasons for vaccine hesitancy.

The study by Hidiroglu et al.,^33^ with 689 health care workers and which aimed to determine the knowledge and opinions on the influenza vaccine and its acceptance in the Medical University of South Carolina, also confirmed that the perceived barriers decreased immunization rates, in addition to evidence from studies by Oria et al.^10^ and Hofmann et al.^31^

A relevant aspect involves some of the reasons presented for not accepting vaccination, such as the fear of adverse effects – pointed out by four of the analyzed studies.^21,22,26,30^ This barrier was corroborated by a literature review comprising various categories of workers^31^ and by the qualitative study by Jaiyeoba et al.,^35^ which investigated the nursing team, attending and resident physicians, and medical students.

We highlight that adverse events can occur after influenza vaccination, but most of them are mild and self-limited. This vaccine has an excellent safety profile, being very effective in these workers, as well as in other categories and age groups, in addition to presenting significant tolerance.^3,4^

### THE TWO NEW DIMENSIONS OF HBM: CUES TO ACTION AND SELF-EFFICACY

The new cues to action and self-efficacy dimensions were added to the HBM in 1994 by Rosenstock et al.^13^ The cues to action dimension can be defined as devices capable of promoting and initiating attitudes for the acceptance of preventive actions (the use of media campaigns, for example).^11^ This dimension was presented in nine articles included in this review; in two studies, no information was found^21,30^ (Table 3).

**Table 3:** Identification of the cues to action and self-efficacy dimensions of the Health Belief Model among the 11 studies

Study	Cues to action	Self-efficacy
A1^20^	The professionals who intended to get vaccinated were positively motivated by the cues to action.	-
A2^21^	-	-
A3^22^	One of the reasons mentioned for vaccination by those who received the vaccine was peer pressure.	-
A4^29^	An association was observed between cues to action and immunization.	-
A5^23^	A strong significant correlation was observed between cues to action and willingness to receive the vaccine.	-
A6^25^	Cues to action were associated with higher chances of getting vaccinated.	-
A7^26^	Encouragement by the physician and supervisor were factors that promoted vaccination.	-
A8^27^	The recommendation of vaccination by the employer was a predictor of vaccine acceptance.	-
A9^24^	Cues to action were predictors of vaccine acceptance.	Vaccinated individuals had higher self-efficacy levels.
A10^30^	-	-
A11^28^	Cues to action contributed to increase immunization rates.	-

Therefore, the authors who evaluated this dimension associated it with influenza immunization, that is, when cues to action were present, the immunization rates were higher.

Other studies also confirmed this association through a systematic review by Corace et al.,^14^ who included various categories of workers, indicating that many cues to action were able to predict influenza vaccine acceptance; the qualitative study by Hidiroglu et al.,^33^ with physicians, nurses, midwives, and sanitarians, presented a relationship between cues to action, such as a medical recommendation, and higher willingness to accept the H1N1 vaccine.

Self-efficacy can be defined as the belief to be sufficiently capable of overcoming difficulties inherent to the preventive attitude.^11^ This dimension is present in only one of the studies reviewed in this study,^24^ indicating that participants who were vaccinated against influenza had higher self-efficacy levels than the unvaccinated ones.

In this regard, two studies also concluded that the self-efficacy dimension of HBM justified the adoption of other preventive measures. The first study, performed by Shewasinad Yehualashet et al.^36^ with a total sample of 683 inhabitants of Ethiopia, aimed to identify predictors of adherence to preventive measures against COVID-19 and showed a significant association between self-efficacy and the level of adherence to safety measures. In the second study, Pribadi &

Devy^37^ investigated 58 young adult women smokers in Indonesia, aiming to analyze the correlation between the intention of quitting smoking a HBM factors, and finding as a result a correlation between willingness to quit smoking and self-efficacy.

Regarding which of the dimensions would be the most important for accepting influenza vaccination, no agreement was found between authors. Two of them agree that the perceived benefits dimension is the most relevant,^20,28^ one author believes it is perceived barriers,^22^ and another reports the perceived benefits for experienced workers and cues to action for unexperienced workers.^24^ The other articles in this review did not draw conclusions on which were the most important dimensions.

Considering the application of HBM in influenza vaccination among health care workers, the authors who expressed general conclusions on this theory stated that the results validated the applicability of the model for understanding vaccination behavior^25^; the theory was able to predict the willingness to receive the vaccine^23^; the levels of the dimensions among vaccinated individuals were significantly higher, except for the barriers category^24^; almost all of the model’s main constructs were significantly associated with the intention (or not) of getting vaccinated^20^; and their results are strongly correlated with HBM principles.^28^

## CONCLUSIONS

The results of this integrative review indicate a correlation between HBM dimensions and influenza vaccination in health care workers. Moreover, the theory as a whole is able to explain and predict vaccine hesitancy or acceptance by workers.

The dimensions most frequently explored by the studies were susceptibility, severity, perceived benefits, and perceived barriers, whereas self-efficacy was the least studied dimension.

Therefore, we recommend the development of studies on vaccination by using the HBM, encompassing specific categories of workers and approaching other types of immunobiologicals, with the perspective of identifying evidence that broadens the possibilities of interventions to reduce vaccine hesitancy among health care workers.
